# Exploring the GP-patient relationship: a historical narration

**DOI:** 10.1136/medhum-2024-012916

**Published:** 2024-10-24

**Authors:** Emma Ladds

**Affiliations:** 1 Nuffield Department of Primary Care Health Sciences, University of Oxford, Oxford, UK

**Keywords:** cultural history, narrative medicine, Primary care

## Abstract

The relationship between patients and their doctor is a fundamental concept—particularly within general practice. Many patients and general practitioners (GPs) have a ‘common-sense’ recognition of the interpersonal connection, usually over time, that makes a relationship meaningful. GPs have consistently striven to emphasise the importance of this connection. While much research has explored the components and influences affecting intersubjective connections, less attention has focused on how the historical, professional, sociopolitical and philosophical contexts have influenced their experience and portrayal. However, recent claims of a crisis in UK general practice resulting from declining relational encounters suggest these are important considerations. In this paper, episodic narration (chronicling) is used to explore five different ages of UK general practice: the emergent period (1815–1948); the expansion of coverage (1949–1965); the professionalisation of general practice (1966–1988); the age of marketisation and neoliberalisation (1989–2004); and the age of technology and fragmentation (2004–present day). A range of sources illustrate micro and macro viewpoints within each period—personal reflections, professional publications, political directives or policies, and representations from the fields of art and literature. This allows for a deeper exploration of contextual influences on the codification and enactment of GP-patient relationships over time and their interpretation and perception. Significant epidemiological and biomedical realities and their respective social interpretation(s), the socioprofessional nature of the GP, that is, their role, societal position and framework of practice, and broader sociopolitical and philosophical factors are explored. Ideological frameworks (from socialism to free market policies and neoliberalism) were particularly important. These determine approaches to funding, service/provider structures, and regulation and governance, which incentivise, enable, or inhibit choices and behaviours among individuals and society, thus impacting the enactment of the GP-patient relationship. If meaningful GP-patient connections are valuable and desirable—as GPs consistently claim they are—we require an honest discussion about the contexts necessary to enable and retain them.

## Introduction

 ”To be modern is to experience personal and social life as a maelstrom, to find one’s world and oneself in perpetual disintegration and renewal, trouble and anguish, ambiguity and contradiction: to be part of a universe in which ‘all that is solid melts into air” (Berman 2010).

Since the early 1800s, general practitioners (GPs) have been immersed in professional, institutional and political flux. Over the centuries, each generation has mourned losses resulting from this relentless change. From Dr John Brown in the 1800s, who claimed that ‘what is now so rare – the old feeling of a family doctor – the familiar, kindly welcoming face, who has presided over births and deaths; the old friend who bears about and keeps sacred, deadly secrets and who knows the kind of stuff his flock is made of; all this stuff is greatly gone’ (Brown 1858-61) to Dr Lindsey Batten in the 1960s, who felt that ‘the guide philosopher and friend part of our job is out – finished’ (Batten 1961) and Dr Clare Gerada, who recently bewailed that ‘unlike 30 years ago all my patients are strangers’ (Gerada 2022), GPs of all eras have clearly valued their longitudinal knowledge of their patients and striven to assert the importance of the human need for connection this affords.

These personal relationships are impacted by numerous factors at different levels. Micro-level examples such as the communication skills or development of trust in consultations occur alongside meso-level factors like the nature of premises, professional activities and practices, or access and care pathways. Many of these have been well studied (Kaba and Sooriakumaran 2007; Ong *et al* 1995; Ridd *et al* 2009). Underpinning these are macro factors such as epidemiological, demographic and technical realities, sociopolitical determinants of professional and cultural practices, and the evolving norms, fashions/fads, philosophies and ideologies of society. These act together to form a complex, interdependent context that impacts relationships themselves, the perspectives of GPs and patients participating in them, and the interpretations of external observers.

Scholars have found the GP-patient relationship challenging to analyse and theorise, particularly the communication and behavioural influences (Ong *et al* 1995, 903–18). Much research has focused on characterising the psychodynamic component with different disciplines emphasising specific aspects (Balint 1957; Garfield 1980; Ridd *et al* 2009; Roter and Hall 1992). Sociologists and psychologists have highlighted social features, that is, good manners, friendliness and honesty (Roter *et al* 1987), or psychotherapeutic elements, for example, empathy, respect, genuineness, unconditional acceptance and warmth (Garfield 1980). While Roter and Hall proposed that ‘talk’ (the verbal and non-verbal interactions) between patient and doctor were crucial in determining behaviours and thus interactions according to the balance of control (Roter and Hall 1992), Balint viewed the relationship as a mutual, psychotherapeutic investment, with both sides contributing capital (Balint 1957).

This range of ontological perspectives highlights that any historiographical commentary of the GP-patient relationship will be subject to the beliefs and opinions of those who portray it. For example, a range of epistemic frameworks and methodological approaches have been used to try and academically define the nature of ‘high-quality’ relationships (Greenhalgh and Iona 2010, 7–29), while many GPs and patients describe a more inductive experience of what this means on a personal level. This latter understanding is often more eloquently expressed within the humanities but may inform academic and professional approaches. As Morland describes in her biographical depiction of a modern GP, ‘a relationship over time fosters familiarity, empathy, understanding, a two-way sense of responsibility, all core ingredients of trust; and this trust then encourages disclosure, improves communication, saves time; which in turn cultivates cooperation and empowerment, reduces anxiety and mistakes, improves the execution of tasks undertaken together […] it all sounds like good common sense’. As she explains, ‘any one of us who has a relationship with anyone else, personal or professional, intuitively knows this stuff’ (Morland 2022, 218–9). This resonates with the value placed by Stange and MacWhinney (Stange *et al* 2001, 286–297), and more recently Lynch *et al* (Lynch *et al* 2021, 638–647), on the pragmatic, practical wisdom and tacit knowledge that traditionally form a cornerstone of general practice.

Although concern about the death of the family doctor is nothing new, a recent ‘maelstrom of modernization’ (Berman 2010, 16), triggered by system pressures and political directives, has resulted in widespread fragmentary and remote working patterns for GPs. The pace and impact of these changes have posed significant challenges to those attempting to develop longitudinal relationships. As Tammes highlights, they are now often resource-intensive and sometimes impossible to achieve (Tammes *et al* 2021, e432–e440). Some commentators have even claimed that the GP-patient relationship is dying (Hoff 2017) and, as Morland reflects, ‘perhaps we have forgotten to expect, or even to want, doctors like her’ (a traditional GP with a high level of continuity) (Morland 2022, 218).

This relational decline and its transactional replacement have detrimentally impacted health outcomes, patient and staff satisfaction, and system efficiency (Ladds *et al* 2023). Sole pursuit of quantitative ratings, targets and evidence-based activities risks devaluing the intangible, *human* aspects of medicine. As Heath highlights, this exploits rationality at the expense of humanity, treating humans as objects rather than subjects (Heath *et al* 2007, 1075–1076). Such a model ignores the reality that a significant proportion of patients in primary care cannot be given a formal diagnostic label yet are still offered care by GPs bearing witness to their everyday struggles. In 1974, Thomas reported this number to be around 40%, describing a subset of ‘temporarily dependent’ patients who engaged in a ‘therapeutic illusion’ with their primary care teams for a short period of time before they stopped consulting (Thomas 1974, 625–626). Such individuals are still prevalent. However, within a more transactional, fragmented and defensive system they face higher risks from unrestricted investigations or treatments offered without the wisdom of *knowing* an individual (Dowrick *et al* 2004, 165–170). Multiple studies emphasise the importance of a longitudinal GP-patient relationship in their management—both to achieve the best outcome for individuals and to contain harms and costs across the system (Olde Hartman *et al* 2017).

These benefits are rarely captured in the narrow, quantitative assessments of healthcare quality in the UK today (Hoff 2017). Commentators have argued that more meaningful appraisals pursue a ‘constructive balance’ between scientific rationality and humanity (Heath 2015). However, their introduction may require a reconsideration of our philosophical approach to healthcare. Generalism (Reeve *et al* 2013; Reeve 2023), clinical pragmatism (Brendel 2007, 311–313) and integration of different disciplines (Nicolescu 2014, 186–199), have all lost out in recent years to innovation and specialisation. Such approaches are participatory, integrative and holistic with a real-world recognition of useful outcomes and uncertainty and restoring their value within the healthcare system may enable high quality, meaningful, person-centred care.

However, such approaches are demanding and effortful, requiring complex skills, knowledge, experience and insight within an enabling context. We might be tempted by the superficial attractiveness of quantitative, reproducible targets but ignoring these human and integrative aspects of medicine comes with significant risk. GPs across the years have endeavoured to illustrate how humans need connection and recognition, which cannot be captured on a form or in numbers. As John Salinsky concludes when reflecting about why he might have helped a patient: ‘it was because I stayed with [patient X] and was able to do that because I was interested in her and moved by her feelings. My interest, my concern for her, and my willingness to share her feelings must, I think, have to do with a feeling of identification which I had with her’ (Salinsky 1987, 105). This existential *need* for reciprocal identification is not unique to GPs and patients, however, it is allowed specific expression and promotion within their relationship. Here we set out to explore the contextual factors that influence this connection, which will be important considerations if we wish to restore ‘care’ to our healthcare systems (Heath and Montori 2023).

## Methods

The value of the historical perspective is well known (Lawrence 1984). One oft-underrated benefit is the capacity offered to highlight temporal touchpoints that have since faded in significance and the negotiated processes whereby certain objects/practices come to, and fade from, our attention (Whitehead and Woods 2022). Traditional academic historical writing frequently attempts to explore such ideas using narrative or monographical forms. The former typically sets out the past in chronological order, depicting events with an interpretation, sense of agency and plot progression (Strawson 2004, 428–452), while the latter is extensive, focused and analytical, detailing a particular subject or time. Although subject to influence ‘by cultural conventions of telling, by the audience and by the social context’ (Denzin 1989, 30) narrative inquiry has provided a useful tool for understanding cause and effect and helping humans make sense of their experience (Mishler 1986, 67–68). As Rousmaniere points out, history is ‘more than tell[ing] a story about the past. History also helps to make meaning about the present’ (Rousmaniere 2004, 50).

The current historical literature contains multiple relevant examples of such approaches. Among many, Irvine Loudon, Geoffrey Rivett and Anne Digby provide chronological, narrative accounts of the events leading up to the formation and evolution of the National Health Service (NHS) and general practice, respectively (Loudon 1986; Rivett 2014; Digby 1999). In contrast, Roger Cooter’s expansive paper exploring the place of doctors in UK politics during the interwar years provides a detailed, monographical analysis of the strategies pursued by the British Medical Association (BMA) to promote the medical profession and its overall influence on the emergence of professional society (Cooter 2004, 59–107). Other historical works lie between the two approaches. For example, Jane Lewis’ analysis of the GP contracts in 1966 and 1990 uses two specific contexts to analyse the different meaning ascribed to the contract at each time and the implications this might hold for future contractual negotiations (Lewis 1997, 895–898), that is, not providing a full narrative chronology but giving a broader perspective that enables useful inference.

In this paper, a chronicling approach has been used, which aligns more closely with the latter example. This aims to minimise the ‘layers of intention and reconstruction’ inherent in the retelling of stories seen in narrative inquiry (Lander 2000) and removes the need for a coherent plot. Instead, particular emphasis is given to the sources used, and process of, capturing and depicting events, ideas and values at different timepoints. In recentring on these ‘representations of witness’, chronicling highlights the meaning behind the very process of witness itself rather than prior or subsequent events (van Hout *et al* 2023). It is hoped this will bring the representations of contextual influences on the GP-patient relationship to the fore, enabling a deeper understanding of the surprisingly consistent assertion about the need for human connection within patient-doctor interactions.

The use of contemporary secondary literature (both factual and creative) as primary literature, and the integration of historical, sociological and psychological perspectives within historical analysis are well established. To take just one recent example, Martin D Moore’s exploration of the development and response to appointment systems within general practice uses a range of different sources. Alongside factual references, numerous papers from humanities and sociology journals, media commentaries, personal accounts, artistic works, oral histories and large societal archives build a chronological representation of the introduction, evolution and influence of the appointment system. Moreover, Moore uses both psychological and sociological theories to enable an understanding of individual-level and societal-level impacts. He describes how, ‘appointments were temporal promises of attention to people in states of illness and uncertainty’ and explains how they ‘reworked long-established social practices and cultural expectations of waiting and care’ (Moore 2002, 206). It is hoped that using a comparable breadth of source material in this paper may help facilitate similar ‘holistic looking’ (Perkins 1988, 122) and thus richer insights.

Selecting from a wealth of source material is always a challenge and is compounded in this paper by the profound subjectivity of ‘relationships’. As a starting point, two well-respected histories of general practice and the NHS were used alongside an appraisal of annual editorials (1953–2024) of the *Journal of the (Royal) College of General Practitioners*/*British Journal of General Practitioners*. It is hoped that as the professional journal for UK GPs, this might ensure identification of particularly salient issues. From these, an iterative snowballing approach identified other relevant materials alongside a concomitant hermeneutic cycle of interpretation (Ricoeur 1976). As Yin has emphasised, ‘every document was written for some specific purpose and some specific audience other than that of the [case] study being done’ (Yin 2003, 87) and as such are representations of ‘the social perception of facts; […] subject to social pressures from the context in which they are obtained’ (Briggs 1986, 13–14). They must therefore be approached with appropriate caution. Moreover, the integral involvement of the researcher necessitates a high degree of reflexivity. Bannister has argued that this enables researchers to ‘choose personally relevant issues of research, to draw on, and make explicit personal experience, [and] to enjoy the wisdom and companionship of your subject’ (Bannister 1981, 199) but it is essential to ensure rigour in this process—both as regards the choice and inclusion of source material and during the act of analytical interpretation. In this paper reflexivity was attempted by triangulating multiple sources to ensure appropriate convergent interpretation alongside disconfirmatory examples; the findings were informally discussed with academics from different backgrounds as well as contemporary GPs; and self-reflective accounts were used throughout the process (Yin 2003, 36).

In subsequent sections, five ages of general practice are described, with a specific focus on ‘NHS’ GP care. The emergence of general practice (1815–1948) is a wide time frame that witnessed the origins of the profession as well as multiple legislative and societal changes that set the stage for NHS GP care. The expansion of coverage (1949–1965) saw the immediate place of general practice within the nationalised service, followed by increasing requirements, under-resourcing and highly variable care. The professionalisation of general practice is explored from 1966 to 1988, when the Royal College of General Practitioners began to flourish, a general practice postgraduate training scheme was developed, and the specialist skills of communication and consulting were recognised. The age of marketisation and neoliberalisation (1989–2004) saw the introduction of the computer, data collection, monitoring, and financial incentivisation while in the age of technology and fragmentation (2004–present day) the progressive use of technology, ready access to (mis)information, and reliance on guidelines and algorithms is resulting in a fragmentary ‘taskification’ that emphasises transactional encounters. In each age, representations of the patient-GP relationship are used to try and understand these interactions, their contextual influences and the meaning ascribed to them.

## The emergence of general practice in the UK (1815–1948)

Although the term ‘General Practitioner’ (GP) was first used in 1809, general practice truly emerged following the Apothecaries Act in 1815 (Gillam 2017). This established the first common licensing arrangements for a GP—an ‘individual qualified in physic and surgery and competent to practice medicine, surgery, midwifery, and pharmacy’ (Clark 1966, 476–9). While professionalising this approach, many argue the Act also condemned GPs to an inferior status compared with their surgeon or physician contemporaries. Thomas Wakley’s proclamation in 1843 that ‘a man so preposterous as to understand both physic and surgery—is fit only to become a subordinate’ (Wakley 1843, 719–22) was a common sentiment. This difference in status hindered the professional development of general practice, delaying the introduction of a royal college, academic departments and formal training schemes. More importantly, it resulted in acrimonious relationships with specialists and a perception that integrative generalism was of lesser value than expert knowledge.

The passing of the Medical Act in 1858, which established the General Medical Council (the regulatory body) and medical register, cemented the difference between physicians, surgeons and GPs. The former took up specialist roles in hospitals, while the latter provided care within the community (Loudon 1986). This gave rise to the aphorism that ‘the physician and surgeon retained the hospital and the GP retained the patient’ (Stevens 1966). This impacted the depth of the relationship GPs formed with their patients as, until recently, most lived and worked in the same community. They therefore knew their patients as shopkeepers, parents or friends as well as through clinical interactions, adding a level of intimacy. This was illustrated by John Berger, who described the ‘social – as distinct from medical – intimacy’ established between the GP and other local men as they laboured together to produce a community garden (Berger and Mohr 1967, 100).

However, living circumstances in the nineteenth and early twentieth centuries were dismal. Industrialisation led to urbanisation and overcrowding resulting in malnutrition, infectious disease and poverty-related ill health. Routine immunisation first became available in the 1940s and 1950s and the first antibiotic, penicillin, was not in routine use until 1946 (Loudon 1986, 101). C W Watts, a GP in the 1930s, recalls how his house calls doubled from 50 to 90 during an epidemic (Watts 1979, 1055–1056). General practice was gruelling. Even during the 1950s the infant mortality rate remained around 36 in 1000 (falling to 8 in 1000 in 1990) and prior to the 1930s 400–500 women died of pregnancy-related conditions per 100 000 births (compared with less than 10 in 100 000 in 1990). Obstetric complications were one of the GP’s most feared encounters (Loudon 1986, 95). As H A Elder describes, reminiscing about the 1920s, ‘many general practitioners were highly skilled obstetricians because they got plenty of practice in dealing with conditions they should never have allowed to occur’. However, he also recounts the emotiveness of such encounters, describing how successful management of a pregnancy, confinement and the postnatal period generated a bond ‘between the doctor and the family that is not easily broken’ (Elder 1964, 336).

Neither physicians nor apothecaries had effective approaches to combat most illnesses. However, the physicians’ fee gained them a reputation for lining their pockets while helping patients into the grave. This was depicted in multiple satirical prints, such as Thomas Rowlandson’s depictions of death mixing up medicines or an angry queue of patients agitating outside a physician’s house (Simpkiss 2021). In turn, this reputation led to mistrust and fear. Many patients turned instead to apothecaries—and later GPs who were too numerous to charge high rates. Paintings and prints from the time highlight these contrasting relationships. For example, Thomas Pelham Hall depicts a patient rejecting the affluently attired physician Oliver Goldsmith in favour of the modestly clad apothecary (Pelham Hall 1856). This contributed to the romanticised notion that the rewards of general practice lay in personal relationships not riches (Ashe 1868)—beneficial in developing the profession’s patient-centred approach but complicit in enabling under-resourcing and undervaluing by policymakers and healthcare leaders.

Family doctors became valued for their knowledge of ‘the hereditary constitutions, habits, and temperaments of their patients’ (Thomson 1837). This enabled a broad clinical and pastoral role, with continuity of care at its core. However, throughout the eighteenth and nineteenth centuries, the rise of laboratory medicine, microscopy, pathology, and their associated methods and physical tools encouraged a more biomedical perspective, in contrast to the contemporary non-rationalistic understanding of disease and illness. Generalists often found a pragmatic approach. For example, Taylor, in his 1954 survey quotes a GP in a busy country practice who sometimes had to ‘cheat’ in patients’ interests and start treatment before knowing exactly what was wrong, that is, common sense conflicted with the *correct* scientific approach (Taylor 1954, 306).

This biomedical shift was also reflected in the language of medical practitioners. In the 1700s, Alexander Morgan uses lay terminology to describe ‘a lad with scalding water thrown in his eye […] cured with lint dressing and warm milk’, while a ‘gentleman of fat habit and always swelled legs was taken with a shivering like the beginning of a fever’ (Morgan 1714-1747, 30–31). However, by 1841 even normal physiological states were subject to biomedical jargon. A 29-year-old woman had ‘a circumscribed tumour extending upwards from her pubes to mid-way between the umbilicus and scrobiculus cordis. This tumour was extremely firm and communicated the sensation of a solid body to the touch’, leading to the conclusion that, ‘the woman was in the 7^th^ or 8^th^ month of utero-gestation’ (Bedingfield 1816, 208). This emphasises and reinforces the significant power imbalance between patient and doctor that persisted for much of the following century and inevitably influenced their relationship. As recently as 1989, despite a greater focus on communication, the title of Byrne and Long’s famous study of the verbal behaviour of GPs, ‘Doctors talking *to* patients’, emphasised the perceived directionality to the interaction that was still regarded as unremarkable (Byrne and Long 1989).

Despite, or perhaps enabled by this power differential, Kleinman has noted how the GP often occupies the land between scientific and lay cultures, helping patients navigate understandings of health, disease and illness to devise meaning (Kleinman 1988). In the face of biomedical inadequacy this may be enacted through broader roles as a guide, philosopher, advisor and friend (Loudon 1984, 347–62), and sometimes by simple commitment or in bearing witness to individuals’ suffering. Artistic representations of GPs during the Romantic period tended to exacerbate and glorify this ideal. Luke Fildes’ infamous painting of Dr Murray observing a sick child (Fildes 1891), Dr Hope in Martineau’s ‘Deerbrook’*,* who strives to improve the public health conditions for his backward population with their ‘folk medicine’ beliefs (Martineau 1884), Dr Woodcourt in ‘Bleak House’ who is similarly devoted to the diseased poor (Dickens 1852) and Trollope’s Dr Thorne (Trollope 1858), all exemplify this hero-doctor trope.

Given the significant shift in views about the biomedical approach that occurred across the nineteenth and early twentieth centuries, it is unsurprising that scientific, modern ideas were initially mistrusted and hospitals regarded with suspicion. In this context old-fashioned, familiar GPs were celebrated as comforting and therapeutic. For example, Mr Mellidew, the new, ‘youngish’ GP from Byron’s ‘Paid in Full’, was valued for having ‘no nonsensical ideas about new theories’ and ‘prescribed very much the same kinds of remedies [as] his late employer’ (Byron 1864-5, 246–7). There was also a recognition of the intimacy and familiarity of the GP’s role. Dickens’ physician in ‘Little Dorritt’ is described as ‘a man who really has an acquaintance with us as we are. Who is admitted to some of us every day with wigs and paints off, hears the wanderings of our minds and sees the undisguised expression of our faces’ (Dickens 1857, 531). Accordance with this virtuous trope and safe familiarity were often more positive contributors to the GP-patient relationship than interpersonal connection, clinical skill or objective outcome. Although this would later change as general scientific literacy increased, it is interesting that as late as 1915, Maylett Smith describes a patient he treated as a locum who left ‘with a gesture of extreme dissatisfaction, disconsolate and aggrieved’ because his ‘unfamiliar’, dark brown medicine (strychnine coloured with brown sugar) was usually ‘the colour of tea’ (Maylett Smith 1984, 23).

Such literary depictions must be interpreted cautiously in the context of romantic Victorian ideals, which frequently attempted to capture and/or promote the nineteenth-century shift from subjectivity to rational empiricism. They also reflect the views of a societal class with the time and money to write. For example, although Dickens’ early life was blighted by poverty, he died a wealthy man and Martineau and Trollope came from comfortable households. Much of the population lived short lives dominated by pain, suffering and low expectations with an equanimity and necessity to relationships. C A Watts describes how, in the face of so much suffering, the family doctor had to be ‘tough’ and ‘cruel to be kind’ although he does recall being ‘conscious stricken’ following the death of a 16-year-old girl from ‘galloping consumption’ revealing that some patients broke through. Thus, for most working-class patients, their relationships with their GP(s) were sparse and transactional with ‘successful treatment […] accepted with gratitude, and the many failures tolerated as inevitable, without rancour or recrimination’ (Watts 1979, 1056).

## Expansion of coverage—GPs thinly spread (1949–1965)

In the early twentieth century, societal status strongly influenced interactions. Having emerged from a tradesman-like role, GPs had campaigned hard to gain regard as qualified, competent, educated generalists with an associated social standing. However, J A Pridham describes how there was great variation, which ranged from those with an Oxbridge education practising among a wealthy clientele to those working in poverty-stricken areas. While the former could achieve wealth and social prestige, the latter ‘considered themselves lucky if by hard work they could earn a competence’ (Pridham 1962, 530). Many doctors displayed a robust or rude attitude to working-class families, which was tolerated or expected. C A Watts’ predecessor infamously whipped the children out of his way as he rode past (Rivett 2014). In contrast, middle-class families with their higher fee were treated more respectfully. Interactions were often imbued with ritual. For example, a napkin, spoon and glass of water would await the doctor, along with their fee, on the mantelpiece. The upper classes simply treated the GP like a superior servant.

For GPs, the patient’s financial situation (and thus their fee) was very important. Entry into a practice was highly competitive and expensive. Most GPs started work with substantial debt. While the introduction of the National Insurance Act in 1911 provided a fixed capitation fee for GPs to cover workers, their families, the unemployed, or chronically sick received no support (National Health Insurance Act 1911). GPs adjusted their fees (and allowable debt) according to their knowledge of a patient’s ability to pay. Retaining patients was a financial necessity. However, H E Elder, a contemporary GP, reports that in his experience, ‘no one who required attention was unable to get it’. He goes on to add that patients suffered not because they could not afford attention but because doctors could offer little effective treatment (Elder 1964, 335). However, Aneurin Bevan, promoting the launch of the NHS, captured the real or perceived impact of fees on the patient-doctor relationship, when he claimed that ‘there is no reason why the whole of the patient-doctor relationship should not be freed from what most of us feel is irrelevant to it – the money factor, the collection of fees or thinking how to pay fees – an aspect of practice that is already distasteful to many practitioners’ (Bevan 1948, 4565).

The passing of the NHS Act in 1946, which granted a family doctor to the entire population, free at the point of use, and the launch of the NHS in 1948 both occurred in a profoundly negative context (Rivett 2014). A rise in Voluntary Hospitals with outpatient departments that competed with GPs for patients, combined with disorganised local authority services, lack of governmental understanding, and the financial destruction of World War II resulted in a ‘horrible and demoralizing sense of disillusion’ among GPs. They returned to ‘the familiar state of isolation with every man for himself in a general atmosphere of cynicism’ (Elder 1964, 337).

It was with this mindset that the profession faced Bevan’s ideological restructuring. Between 1946 and 1948, the BMA campaigned vehemently against the terms being offered to doctors. One former chairman commented, ‘I have examined the Bill [the NHS Act] and it looks to me uncommonly like the first step, and a big one, to National Socialism as practised in Germany’, while a well-publicised BMA poll claimed only 4734 doctors, out of the 45 148 polled, were in favour of the NHS (Broxton 2017). Bevan was no better, claiming that the BMA leadership were ‘raucous voiced’ and ‘politically poisoned’, engaging in organised sabotage of the NHS Act (Bevan 1948a).

This sour, tension-filled, acrimonious environment inevitably impacted the patient-GP relationship. Even doctors who did not oppose the principle of the NHS had practical concerns about its implementation. A contemporary *BMJ* editorial highlighted the ‘dogma, timidity, lack of incentive, administrative hypertrophy, stereotyped procedures, and lack of intellectual freedom’ feared within a state medical service and implored doctors to ‘exercise much tact and patience over the coming months if patients demand – as some of them will – the impossible’. It also highlighted that GPs were becoming social servants linked to their patients’ and communities’ circumstances and expectations, with Pridham noting the ‘frequent strain’ this put on the patient-doctor relationship (Pridham 1962, 534).

The NHS Act inevitably introduced stresses for general practice. Consultation rates increased as patients presented with undiagnosed issues or uncertainty about symptoms. Moreover, their newly established ‘gatekeeper’ role further increased workload. A lack of funding, absence of incentives to improve standards, and an overworked, short-staffed workforce resulted in damning conclusions from two 1950s’ surveys. Collings reported that ‘the overall state of general practice in England is bad and still deteriorating’ (Collings 1950, 547), while Taylor reflected that ‘about one quarter of general practitioners are very good indeed. About one half are good, sound, and reliable and would not hesitate to call them in for one’s family…the remaining quarter are less satisfactory [with] a final twentieth for whom it is difficult to find any excuse’ (Taylor 1954, 8). Part of the trouble was that general practice was still seen as a cottage industry with no specific vocational training or recognition of a GP’s professional value (Standing Medical Advisory Committee 1963). Indeed, Lord Moran infamously claimed that GPs were failed specialists, referring to, ‘a ladder which people are constantly falling off’ (Curwen 1964, 38).

The gaze of the GP was also shifting. Elder reminisced how in the 1920s ‘we knew so little and understood so little’, with ‘a splodge of blood coughed onto a handkerchief produc[ing] a shock of fear and horror’, in case it represented tuberculosis, and how ‘it was common to stand by helpless and watch a strapping young man die in six days without being able to influence the disease [pneumonia] in the slightest’ (Elder 1964, 331). However, following the introduction of penicillin in 1940 and subsequent roll-out of mass vaccination, the GP was equipped with an ever-increasing armoury. Accurate diagnosis gained a practical—rather than just academic—importance and in 1953 the GMC decreed that all doctors must spend a year training in hospital before practising independently. The GP’s approach became increasingly biomedical with a focus on the physical and a pejorative stigmatisation of the psychological. Taylor emphasised how, ‘the better the doctor, the less often does he diagnose neurosis’ and described how he never revealed a patient’s blood pressure to them because, ‘once a patient knows he or she has hypertension, symptoms multiply enormously and misery grows’ (Taylor 1954, 417). Thus, there was little unique or distinct about the GP’s role. It was hospital medicine (of highly variable quality) in a community setting.

## The professionalisation of general practice (1966–1988)

A fundamental question still plagues general practice: What *is* it? What is the essence of general practice and the GP’s role? McWhinney and Starfield emphasised the importance of treating the individual patient in their family, community, and cultural context and highlighted four key attributes of the profession: first contact access; a long-term person focus (ie, continuity); comprehensiveness; and coordination (McWhinney 1997; Starfield 1998). Emphasis has also been placed on the integrative nature of the role (Heyrman and Spreeuwenbergh 1987) and its wider responsibility to an organisation and healthcare system (Olesen *et al* 2000). However, as recently as 2021, an entire edition of the *British Journal of General Practice* explored the existential crisis in UK general practice (Lawson 2021). Summaries of *what* general practice *does* remain high-level rather than clear and directional, for example: ‘[general practice] *restore*[s] *order to the chaos of symptoms so people can contribute to the health and wealth of their nation’* (Hay 2021, 347) and it is frequently pushed into ‘*defining itself at its own margins* [i.e. what it is NOT] *leaving its very centre, its specific priorities, unfathomed by both critics and spokesmen’* (Rudebeck 1992). This lack of clarity has contributed to the ongoing challenge of trying to promote the profession’s value.

Defining and developing a professional identity is a sociological enterprise. It relies on the establishment of values and practices, perpetuated and honed through discourse, practice, education, and training. It was not until 1952 that a College of General Practitioners was founded. This granted some formal recognition to the profession and facilitated a ‘language’ of general practice that enabled further professional activities (McWhinney 1966). The introduction of the ‘Membership of the (Royal) College of General Practitioners’ examination in 1965 and postgraduate ‘vocational training scheme’ (VTS) in 1976 highlighted the unique skill set GPs possessed. Prior to this, generalist GPs had undertaken limited aspects of specialist training, resulting in a lower professional regard and more biomedical patient interactions. However, in 1972, *The Future General Practitioner: Learning and Teaching* (Royal College of General Practioners 1972), captured a growing interest in psychodynamic approaches (Balint 1957), psychological components of illness (McWhinney 1966), and communication and consultation skills (Byrne and Long 1989; Browne and Freeling 1976; Neighbour 1987). It formed the basis of the GP VTS and heralded a transition point in the GP’s gaze.

Although initially written in the 1950s, Balint’s work did not take root until the Family Doctor Charter of 1966 reinvigorated the profession with more resources. Balint reframed the language of general practice, distinguishing it from hospital medicine, and introduced the idea of holistic generalism as a specialty (Reeve 2023). Using powerful metaphors including the ‘drug doctor’ and ‘mutual investment company’ (Balint 1957, 5, 133), Balint’s approach encouraged practical changes like the introduction of personal patient lists and provided a rationale for continuity of care (Balint 1957). A wealth of influential publications subsequently highlighted the ‘art’ of general practice (Morrell 1965), celebrating its unique role (McWhinney 1966), and promoting research into the GP-patient relationship, communication and the consultation (Byrne and Long 1989; Pendleton 1984; Stott and Davis 1979). With its own research base, general practice moved away from its cottage industry roots, with GPs becoming experts in the consultation and specialists in providing personal, holistic, community-centred, longitudinal care for patients (Pereira Gray 1979; Hjortdahl 1992).

The significance of this connection between patient and GP was eloquently captured in Berger and Mohr’s depiction of Dr John Eksell (Dr Sassell). His longitudinal knowledge of his patients and commitment to them allowed him to meet the ‘deep but unformulated expectation of the sick for a sense of fraternity’, because he can ‘recognize’ them, in a way that would not be possible with a superficial understanding (Berger and Mohr 1967, 71). Such a relationship gives a powerful meaning to simple acts and conversations. For example, sharing a cup of tea with an old man awaiting the death of his wife becomes a sign of compassion and comfort because Eksell knows the value of simply being there to mark the significance of the moment (Berger and Mohr 1967, 35).

While clinical knowledge and practical skill are essential, connecting on this level adds a depth to a GP’s practice that enables them to truly answer the needs of their patients. Moreover, it offers a personal satisfaction to the GP on a human level and by enabling a higher quality of practice. For example, writing about the GP now working in Eksell’s former practice, Polly Morland describes how she ‘crav[es] continuity professionally’ as ‘the better she [knows] her patients the more this ground[s] her clinical practice in something that [feels] warm and human, and that this, in turn, enhances the care a doctor is able to give’. She goes on to give an example of the doctor detecting a serious condition in a young, ‘well’ patient because, ‘knowing him as she does, he [didn’t] look right’ (Morland 2022, 110). The evidence bears out this out. Increased continuity is associated with improved medication adherence and diabetes control (O’Connor *et al* 1998), reduced mortality rates (Pereira Gray *et al* 2018), and higher patient and GP satisfaction (Saultz and Albedaiwi 2004). Given these values, there is some debate about the biomedical orientation of the current GP VTS curriculum (Royal College of General Practioners 2023), which arguably underemphasises the personal qualities, integrative wisdom and humanism experienced in high-quality general practice (Greenhalgh 2008; Heath 1997; Lynch *et al* 2021).

General practice has traditionally followed an apprenticeship training model. Therefore, as single-handed practitioners moved to partnerships and the VTS scheme introduced a ‘trainer-trainee’ structure, the process of transferring and reinforcing these approaches became situated within communities of practice. Gabbay and Le May describe how practitioners actively draw on this collective culture, knowledge and practice to develop ‘Mindlines’, that is, using ‘evidence from a wide range of sources melded with tacit knowledge through experience and continual learning that become internalised as a clinician’s personal guide’ (Gabbay and Le May 2016, 402), which allows them to apply this collective wisdom to individual patients. Eksell’s successor illustrates this in her reflection of his impact on her practice, ‘I’d come with a fairly narrow, restrictive medical training, very structured and hierarchical, and all of a sudden I arrived with John and I’m thinking, ‘Blimey, can you do that?’ It left me floundering to begin with, but what he taught me was that caring for people and the art of medicine was about much more than just sitting there and giving people pills or cutting them open and sewing them back up again. It truly was an art’ (Morland 2022, 68). For several decades this holistic art of general practice was celebrated and flourished.

## The age of marketisation and neoliberalisation (1989–2004)

In 1988, Julian Tudor Hart published, ‘A new kind of doctor’, highlighting important health inequalities and advocating social justice in addressing them (Tudor-Hart 1988). Contrary to the prevailing narrative, Tudor Hart emphasised the value of recording quantitative data to identify such disparities. Although commendable for enabling targeted upstream population-level health interventions, this approach also initiated a neoliberal shift that culminated in significant disruption of the dyadic GP-patient relationship.

Tudor Hart’s publication captured the profound epidemiological shift that occurred over the twentieth century. Thanks to public health measures and medical advances, infectious diseases and obstetric complications became infrequent, treatable events and attention shifted towards chronic conditions—cardiovascular disease, cancer and diabetes. This resulted in a surge of clinical trials, often supported by the pharmaceutical industry, to explore their management. Thus, the shadow of private finance crept closer to healthcare.

On a population level, the improvement in health outcomes was undeniable. Between 1980 and 2013 UK mortality from cardiovascular disease declined by 68% (Bhatnagar *et al* 2016). However, the evidence-based guidelines produced from such trials, combined with concepts like ‘QUALYS’ (quality adjusted life years), resulted in a monetary worth being placed on a particular course of action. This added a value judgement (largely financial) about how a GP ought to act, without necessarily considering a specific patient, GP or the relationship they had formed. Peter Toon has described the tensions between these biomedical, humanist and preventative/public health frameworks, which are underpinned by different philosophical assumptions about ‘good’ practice (Toon 1994, viii). Sociopolitical or financial realities, professional altruism and a practitioner’s individual values mediate this strain. Thus, in the UK, the day-to-day reality of general practice is significantly influenced by the political ideology of the incumbent government, which determines what will be funded or incentivised, affecting public perceptions and expectations.

In 1989, this was borne out in ‘Working For Patients’, a series of reforms introduced by the Conservative government. Although ostensibly aiming for cost efficiencies and improved outcomes, the changes increased bureaucratic control and fragmentation of care. The ‘Patient’s Charter’ in 1991 emphasised the patient as a consumer, enshrining them with rights and expectations (Department of Health 1991), while further changes throughout the 1990s emphasised patient choice. Alternative providers like community pharmacies or private screening companies were encouraged to offer competitive services to the NHS and care became increasingly incoherent and transactional (Reynolds 2018, 374–5).

The introduction of the computer in the late 1970s/early 1980s also had a disruptive influence. In GP consultations, for example, improvements in information exchange were counterbalanced by reduced patient-centredness (Shachak and Reis 2009) with patients becoming synchronised to, or limited by, the rhythms of the keyboard (Greatbatch *et al* 1995). GPs generally spent increasing amounts of time on computer-related tasks and less on interpersonal dialogue (Margalit *et al* 2006) and consultations became observable, measurable and reportable to third parties (Garfinkel 1967).

This systematic recording had far-reaching consequences. In a laudable attempt to share best practice and encourage its uptake, the National Service Frameworks of the 1990s and their 2004 successor, the Quality Outcomes Framework, used such data to incentivise evidence-based prescribing. However, this resulted in daily activities primarily focused on the achievements of central targets (Giddens 1991), promoting a risk prediction approach to healthcare, overcategorisation and medicalisation and a belief that all disease could be prevented or controlled. The concept of illness (and health) moved from the individual level to the population level, reducing patients to statistics rather than lived-in subjects and encouraging transactional encounters with quantifiable outcomes.

The rapid expansion of such accountability systems complexified GP-patient encounters. Now GPs had to consider coding, target alerts, prescribing guidelines, risk prediction models, etc, alongside the patient. As Giddens emphasises, there is an assumption that the technical knowledge within these systems has an independent validity and authority to override situational factors. This results in a primacy for the rules (or computer) (Giddens 1991) and a level of micromanagement that devalues the application of practical, integrated and contextually dependent wisdom central to general practice (Lynch *et al* 2021). Moreover, it demonstrates a lack of professional trust and respect for GPs and undermines their relationships with patients (Whitaker 2023b, 54; Gubb 2009).

Most GPs, when grounded in professional communities of practice, intuitively *know* what is expected of them. They understand what practical virtues will allow them to flourish (Toon 2014) and what high quality care feels like—even if it is difficult to measure. Medicine’s internal goods are broadly clustered around caring, curing, and comforting and Held has described the importance of emotion and interdependency in their expression, alongside reason and rationalistic deductions (Held 2006). Moreover, Parsons has emphasised how aligning this commitment to the ethics and values of a community can counterbalance the impact of bureaucracy, individualism, and market forces on health inequalities and outcomes (Parsons 1964). Regulatory or system factors that inhibit professionals in such activities result in moral injury and demoralisation, impairing relationships with patients and quality of care.

## The age of technology and fragmentation (2004–2023)

From 2004, such inhibitory system factors became increasingly intrusive. A new GP contract and subsequent policy directives imposed numerous structural changes, processes, and incentives lacking normative values and contextual sensitivity that profoundly disrupted general practice. For example, GPs were able to opt out of providing out-of-hours care and a salaried GP role was introduced. These changes undermined the continuous personal responsibility and ownership central to the GP-patient relationship and Tammes has documented the subsequent decline in relational continuity (Tammes *et al* 2021). As Scott has described, this caring commitment is central to healing relationships, with the ‘promise not to abandon the patient, even if pills and technology have little left to offer’. He quotes one patient describing how his physician ‘never gave up on me. And that means a lot’ (Scott *et al* 2008, 319). These contractual changes have understandably led to claims that GPs are ‘operating like taxi ranks – impersonal and industrial’, with nobody taking responsibility or caring and many patients feeling ‘fobbed off’ (Whitaker 2023b).

An acceleration of neoliberal and market-oriented interventions further undermined continuity and relationships. For example, the Extended Hours initiative, which incentivised GPs to provide appointments outside working hours, stretched the existing workforce further, contributing to burn-out and poor retention, and paradoxically worsening access (Wellings and Baird 2017). Similarly, the 2019 Additional Roles Reimbursement Scheme, which funded non-GP clinically associated roles in primary care including clinical pharmacists, paramedics and physician associates, has added work and anxiety for GPs. While helpful for some straightforward tasks, for example: medication reviews, such individuals have a range of backgrounds and experience and often require supervision and training. Hence GPs have less time for their own patients, which further dilutes their relationships (Baird *et al* 2020).

The rapid expansion of technology and algorithmic systems has also added confusion. For example, the nationwide non-emergency phone line (NHS 111), introduced in 2013 to cope with out-of-hours demand, is manned by non-clinical call operators. The actions of these individuals are standardised and constrained by structures across the network, thus they are unable to make contingent decisions in specific cases. One such structure is the triaging software, which is necessarily biased towards risk-averse decisions. This results in perverse, inefficient outcomes, causing confusion and frustration for patients and physicians (Turner *et al* 2013). For example, Whitaker describes a father who calls to ask whether his child can attend school having vomited from travel sickness. To their surprise the 111 call-handler sends an emergency ambulance to assess the child—‘vomiting’ in a child triggers an algorithmic risk-averse response. This is an illogical, inappropriate use of resources easily avoided by a meaningful encounter with a clinician (Whitaker 2023b, 122).

The impact of the internet has also been profound. In the late 1800s, scientific rationalism was disseminated slowly through formal publications, textbooks and professional communities, maintaining the split between professionals and the population. However, the internet and social media have democratised instantly available (mis)information, disrupting the power dynamics within the patient-GP relationship and adding multiple layers of complexity. While ready access to previously privileged ‘professional’ information may enable greater equity and patient control within the relationship (Eysenbach 2000), Hart *et al* have demonstrated that for many patients—and indeed some practitioners—the information within the internet takes on a symbolic power that can dominate an encounter (Hart *et al* 2004). Moreover, within the confines of a resource-constrained system, patients may be frustrated if they are unable to access referrals or treatments the internet has encouraged them to believe they *need*. The GP may become the visible barrier standing in their way.

Online patient support groups and social media can enable a similarly powerful patient voice and collective activism. While often positive, this may also challenge the professional narrative—as seen recently with the emergence of long COVID-19 (Callard and Perego 2021)—with potentially profound consequences. For example, during the recent pandemic, misinformation about COVID-19 and vaccines was spread on social media platforms at such a rate that the WHO coined the phrase ‘infodemic’ (WHO 2021). Wilson and Wiysonge have demonstrated that social media use is associated with vaccine hesitancy and lower vaccination rates (Wilson and Wiysonge 2020), demonstrating the real impact of this false or misleading information. Such misinformation both reflects, and contributes to, a wider erosion of trust in healthcare (Strully *et al* 2021). However, interestingly, encounters with trusted individuals (often GPs) have proved essential in overcoming such societally driven hesitancy (Wilkinson 2024), demonstrating the persistent value of interpersonal trust, even in the ‘digital age’.

Kessler has identified multiple influences for interpersonal trust, including, among others, ability-based characteristics and personal features—such as demographics or personality traits (Kessler *et al* 2017). Greater proximity has also been associated with higher levels (Aubert and Kelsey 2003). In contrast, the disembodied and distanciated nature of social media results in a sense of deindividuation, that is, individuals become immersed in a group, unable to meaningfully perceive the emotions or feelings of others (Kiesler *et al* 1984). This frequently results in disinhibition and greater tendency for incivility, which manifests as online ‘trolling’ or physical abuse against mob targets or those with opposing views (Duggan 2017). In recent years this has been exemplified by the rising anti-GP rhetoric, both in traditional and social media sources, often centring around emotive issues like patient access or consultation type (Mroz *et al* 2022). Such claims—frequently shown to be factually incorrect—have fuelled online abuse and physical assaults of GPs and practices (Young 2023; Tonkin 2021). Inevitably this has a profound impact on individual relationships and morale more widely.

Ready access to the internet also promotes a reliance on algorithms and guidelines among patients and GPs that reinforces the biomedical lens. Patients with vague, non-textbook presentations risk being simplified and made to fit these models to cope with their inherent uncertainty. Moreover, patients can increasingly choose to pay for an online ‘solution’ for their symptoms, which contributes to unrealistic expectations and the seductive idea that we can offer technical solutions to the insoluble challenges of ageing, death and loss (Shapin and Martyn 2000). Without a holistic integration, they may endure multiple specialist referrals and investigations to ensure adherence to each symptom-driven guideline—or until they get the ‘answer’ they believe they need. In a culture of defensive practice, the risks and potential harms of overinvestigation are frequently dominated by the fear of missing something. Moreover, Heath has described how the ‘map of biomedical science only roughly matches the territory of human suffering’ (Heath 2016, 2). In focusing on the former, we risk overlooking the latter, which has led to concerns that our modern system faces a ‘crisis in care’ (Heath and Montori 2023).

Quoting Rebecca Solnit reflecting on the contrast between the roses that George Orwell planted while writing about the bleakest, darkest times (Solnit 2021), Heath has used the Suffragettes’ slogan of ‘bread and roses’ to explain what humans want from healthcare: ‘Bread is sustenance and therefore life; roses are courage and hope, curiosity and joy, and all that makes a life worth living. Bread is biology; roses are biography. Bread is transactional and technocratic; roses are relational. Bread is science; roses are care, kindness and love’ (Heath and Montori 2023). A system that fails to recognise the importance of both will never be able to offer the care those within it so urgently need.

## Conclusion

No wonder that among this wealth of complexity it is difficult for individual GPs and their patients to define and retain value in their relationships. From the apothecary’s affordable remedies to the intimate familiarity and transactional pragmatism of the Victorian age; biomedical rationalism to psychodynamic relationality; and from cottage-industry to technical profession GPs have striven to retain a recognition of the human need for relationships that enable connection and meaning making.

These relationships have been enacted very differently according to their contextual roots. Nevertheless, their core value has remained noticeably consistent. It would be easy, in the technological, fragmented, uncaring contemporary world to assume as Berman describes that, ‘modern men and women may well prefer […] the sort of individualism that scorns and fears connections with other people as threats to the self’s integrity rather than the sort of collectivism that seeks to submerge itself in a social role’ (Berman 2010, 110). And yet, Marx’s recognition that, ‘only in community with others has the individual the means of cultivating his gifts in all direction’ (Marx and Frederick 1845-6) retains an important resonance. Relationships still matter. Many GPs still know this—and believe their patients know it too.

## Data Availability

No data are available.
